# Respiratory Syncytial Virus Pediatric Hospitalization in the COVID-19 Era

**DOI:** 10.3390/ijerph192315455

**Published:** 2022-11-22

**Authors:** Elena Bozzola, Sarah Barni, Alberto Villani

**Affiliations:** Pediatric Unit, IRCCS Bambino Gesù Children Hospital, 00100 Rome, Italy

**Keywords:** respiratory syncytial virus, children, hospitalization, COVID-19

## Abstract

Respiratory syncytial virus (RSV) globally affects the population, mainly young children, potentially causing hospitalization. During the COVID-19 pandemic, non-pharmacological measures interfered with the circulation of most respiratory viruses. Then, with the discontinuation of restrictive measures, a new scenario appeared. With this scoping review, we want to globally explore whether the RSV paediatric hospitalization rate was influenced by COVID-19. This scoping review was performed according to PRISMA guidelines on PubMed using the Mesh terms “Respiratory Syncytial Viruses”[Mesh] AND “COVID-19”[Mesh] OR “SARS-CoV-2”[Mesh]. Among them, we identified studies pertaining to children and adolescents up to 18 years old hospitalized for RSV, including 18 records in the revision. With the onset of the COVID-19 pandemic, a drastic reduction in RSV hospitalization among the pediatric population in 2020–2021 season has been observed in the Northern and Southern hemispheres. After the relaxing of restrictive measures, unexpected outbreaks happened, leading to increased hospitalization and occupation of pediatric intensive care units.

## 1. Introduction

Respiratory syncytial virus (RSV) is the leading cause of hospitalization for respiratory tract infections in healthy infants and young children [1]. Almost all children are infected by the age of two years, mostly within the first year of life. As with other respiratory viruses, RSV is spread by droplets from airways, requiring close contact. Transmission may also occur by contact in a close environment of infected people. Clinical manifestations may vary from mild disease to a severe form of infection which requires hospitalization and eventually pediatric intensive care unit (PICU) admission [2,3].

The main concern regards severe forms of viral infection which require hospitalization [2,3].

A severe RSV form may require a prolonged hospitalization, mostly in the youngest, with a negative economic impact on the society [2].

Moreover, RSV infections are linked with an increased risk for long-term sequelae, including recurring wheezing or asthma [2].

There is not a vaccine that may protect the whole population against RSV. The infection may be prevented in toddlers with a high risk of complications, including premature children and children with severe pulmonary or cardiac comorbidities, by palivizumab, a monoclonal antibody against RSV. At present, there are no effective preventative therapies for all the other infants. Most infants and children infected with acute lower respiratory illness are otherwise healthy, suggesting that preventive measures for otherwise healthy infants should be required, including pediatric, maternal vaccines and monoclonal antibodies [1,2,3].

In December 2019, the onset of SARS-CoV-2 causing COVID-19 rapidly evolved into a world pandemic, modifying the lifestyle of people worldwide. Non-pharmacological interventions (NPIs), including protective masks and lockdowns, were applied differently based on national decisions, interfering with the circulation of most respiratory viruses [4,5].

The aim of this study is to globally explore how the RSV pediatric hospitalization rate was influenced by the COVID-19 pandemic.

## 2. Materials and Methods

This scoping review has been performed according to the PRISMA Extension guidelines for Scoping Reviews [6].

An electronic search was undertaken on PubMed database on 12 September 2022. To avoid missing results that may be of note for our revision study, constructing our search in PubMed, we used all of the important concepts from our basic clinical question, avoiding unnecessary filters. The key words used to perform the research were: “Respiratory Syncytial Viruses” and “COVID-19” or “SARS-CoV-2”. They have been used as Mesh Terms (“Respiratory Syncytial Viruses”[Mesh]) AND (“COVID-19”[Mesh] OR “SARS-CoV-2”[Mesh]).

Studies were identified as eligible for this scoping review if they met the following inclusion criteria:

- Full-length articles or reviews;
- Pertaining to children and adolescents up to 18 years old;
- Comparison of the rate of hospitalization for RSV before, within, and after COVID-19 pandemic.

The exclusion criteria were:

- Reports not in English;
- Not pertinent to the field of investigation;
- Involving adults (>18 years);
- Involving outpatients.

To reduce errors and bias, two researchers independently analyzed titles and abstracts to exclude distinctly irrelevant articles. Finally, the eligibility of the articles was confirmed by evaluating the full text. Disagreements regarding inclusion/exclusion were settled by discussion between the researchers.

Furthermore, in order to find all the pertinent articles, the reference lists of chosen articles were also analyzed.

Afterwards, data were compiled in a Microsoft Excel spreadsheet to calculate frequencies and percentages of the problems related to RSV.

The review included the following key phases:

- Stage 1: identifying key words appropriate for the research;
- Stage 2: extrapolating from PubMed the literature found with key words;
- Stage 3: initial screening of titles and abstracts;
- Stage 4: retrieval and screening of the full texts;
- Stage 5: retrieving articles from references of selected reports;
- Stage 6: collating, summarizing, and reporting the results.

## 3. Results

The search on the selected database produced n. 151 among articles and reviews. All the documents have been reviewed for relevance and eligibility. No duplications or foreign language works were identified.

According to PRISMA guidelines, of the identified items, all abstracts were analyzed, and 76 records were excluded, as they dealt with other topics irrelevant to this review (e.g., laboratory analysis and research, vaccines and therapy, fragile patients, case reports, coinfections etc.). So, 75 records were to be analyzed by reading their full-length articles. All articles have been retrieved and assessed for eligibility, and then 57 were excluded because 22 included an adult population in their analyses, 26 included outpatients, and 10 reported generic information with no data. Finally, one relevant report found cited in another study was added to this research.

**Figure 1:**
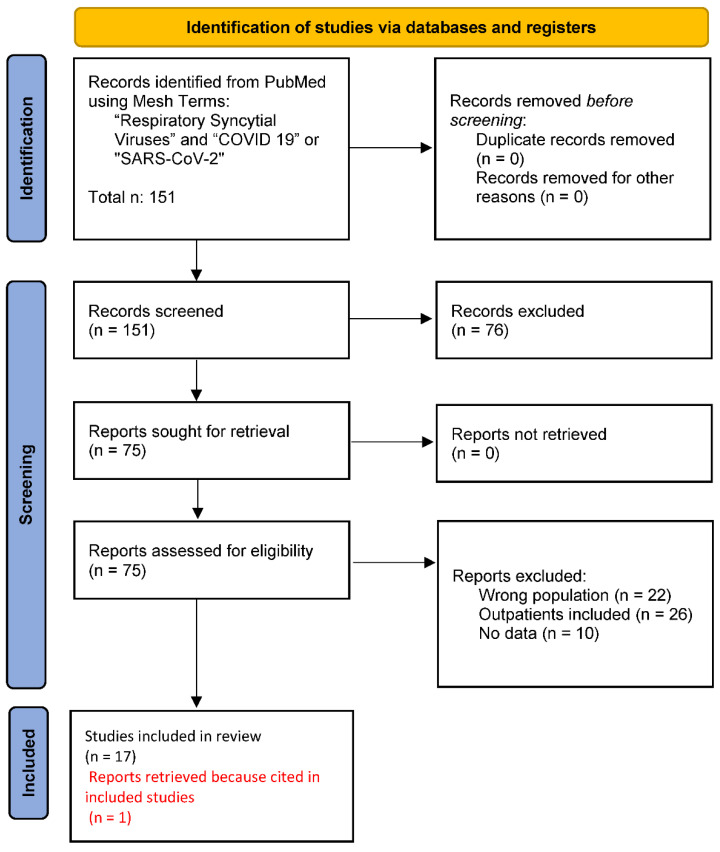
Flow chart of the selected process, according to PRISMA guidelines: 151 reports were selected from PubMed, none of which were removed for duplication nor for foreign language. Seventy-six records were excluded by reading their abstracts because they dealt with the wrong topic. Seventy-five reports were analyzed full-length, fifty-seven of which were excluded: twenty-two dealt with the wrong population, twenty-six included outpatients, and ten had no data reported. One relevant report was found cited in another study and, therefore, was added to this research.

In conclusion, 18 records were included in the revision [7,8,9,10,11,12,13,14,15,16,17,18,19,20,21,22,23,24]. The included studies analyze RSV pediatric hospitalization in COVID-19 era in Asia [5], America [2], Europe [7], and Oceania [4]. Diagram 1 presents the flow chart of the selection process, adapted from PRISMA guidelines [25] (Figure 1).

The included reports had been analyzed and the main highlights are presented in the table [7,8,9,10,11,12,13,14,15,16,17,18,19,20,21,22,23,24] (Table 1).

Regardless of the continent in which the study was performed, most reports agreed on a decreased occurrence of RSV pediatric hospitalization during the COVID-19 pandemic period compared to previous seasons [7,8,9,10,11,12,13,14,15,16,17,19,20,21,22,23,24]. Later, many countries experienced delayed RSV peaks, leading to unseasonal outbreaks and hospitalizations [7,8,9,10,13,14,15,16,19,20,21,22,23]. Of note, among inpatients, RSV disease has been described as more severe than in the past and may have more frequently required oxygen supplementation or intensive care assistance [7,8,9,19,20]. Some studies reported an increase in the median age of hospitalized children compared to previous seasons [14,19,20,23].

## 4. Discussion

With the onset of the COVID-19 pandemic, public health interventions, including school and business closure, stay-at-home orders, and lockdowns, globally reduced individuals’ interactions. According to the European Centre for Disease Prevention and Control surveillance atlas data, the circulation of RSV stopped immediately after NPIs were introduced to control SARS-CoV-2 circulation in February–March 2020, with just sporadic epidemics observed in France and Iceland during the 2020/21 winter [26].

NPI influenced not only SARS-CoV-2, but also other respiratory viruses’ circulation.

A drastic reduction in respiratory viruses’ circulation, including RSV, and hospitalization in the 2020–2021 season have been observed in both the Northern and Southern hemispheres [7,9,10,11,21]. The rate of PICU admission, as well as of ward hospitalization, decreased in pediatric patients affected by RSV. In most cases, the decrease was significant, as in Torres-Fernandez et al.’s study, in which the monthly median hospitalization moved from 59 in 2018 to 0.5 in 2020 and the PICU admission from 6 to 0 in the retrospective multicenter study [16].

These results may suggest that the series of preventive and control measures against SARS-CoV-2 were also effective in stopping the spread of RSV [10,15,16].

In contrast to these results, Li et al. reported an increased RSV incidence during the COVID-19 period (September–December 2020) compared to the pre-pandemic one (September–December 2019) among hospitalized children for acute respiratory infection (20.1% vs. 6.6%). A possible explanation for this may be that enrolled patients were aged less than 2 years and were not likely to use protective masks [18].

Following the initiation of mass COVID-19 vaccination, COVID-19 mitigation practices such as physical distancing, home isolation, and the cessation of global travel have become less stringent with the relaxation of public health measures. In most countries, resurgences of RSV infection were observed after an absence for more than one year and it varied in intensity [10,13,15]. An Australian report which compared the peak months for 2019 (July) and 2020 (December) even reported a 2.5-fold higher incidence of RSV-positive admissions in December 2020 compared to July 2019 [19].

The peak RSV epidemic was delayed by 2 to 9 months compared to those in the recent pre-COVID-19 period, leading to unseasonal outbreaks [7,9,13,15,23]. Multiple factors, including lifted travel restrictions and the resumption of socioeconomic activities, may have triggered this unusual epidemic. Differences in the re-emergence of RSV may be connected to national policy, specifically how each country has dealt with COVID-19 and, particularly, how they managed to use NPI to mitigate the pandemic [9,23].

Children who were not exposed to RSV in 2020–2021 were more susceptible to viruses and might have rapidly transmitted the viruses to each other. The immunity debt is of particular concern for RSV, for which immunity is obtained through exposure, except for a small percentage of children eligible to palivizumab, a monoclonal antibody [2,17].

Researchers underlined differences regarding the magnitude of the RSV outbreak, the severity of the RSV disease, and the virus pattern [7,8,9,10,14,17]. As for strain, researchers found a prevalence of RSV B in the season of 2021/2022, as well as in the seasons of 2017/2018 and 2019/2020 [7]. Nevertheless, among hospitalized children, with RSV B infection in 2021/2022, the percentage of those requiring respiratory support was significantly higher compared with the previous seasons (32.5% vs. 14.7% in 2017/2018 and 19.4% in 2019/2020) [7]. According to age group, the percentage of patients requiring respiratory support was significantly higher in 3–24-month-old children in 2021/2022 season (19.2%) than in the previous ones (1.8% in 2017/2018 and 6% in 2019/2020) [7].

In New Zealand, in 2021, a three-times higher hospitalization incidence in children aged 0–4 years than the average of peaks in 2015–19 was noted. However, the most concerning result is the increase in PICU occupation, which was 2.8 times higher than the average of peaks in the previous seasons [17]. An increased severity of the infection has been confirmed by Movva et al., who registered an increased PICU admission (69%) of infants hospitalized for RSV compared to pre-epidemic period (60%) [8]. A more severe course of the disease may be explained by a reduced immunity from a lack of previous exposure in 2020.

By contrast, some authors underlined a reduced need of supplementary oxygen in pediatric patients hospitalized for RSV and less frequent requirement of admission to the intensive care unit and the use of non-invasive ventilation [14,20]. A possible explanation may be the age of patients included in the study. Compared with 2014–2017 seasons, patients with RSV infection were older during the 2020–2021 season (24.5 vs. 13.2 months, *p* < 0.001), with a higher proportion of patients aged >2 years (41/80 vs. 7/48, *p* < 0.001) in Lee at al.’s study [14]. An increase in RSV cases and patients’ age may be linked to a greater cohort of RSV-naïve patients. Considering toddlers, French reports underlined a fewer number of infants aged less than 6 months compared to the previous seasonal period (41.3% versus 56.6%, *p* < 0.0001) [20]. The older median age of children hospitalized with RSV may explain the smaller proportion of severe infections, as suggested by the shorter length of stay in hospital and the lower need of admission to PICU in 2020/2021 compared to previous outbreaks [20]. An Australian study also confirmed that the age-specific incidence was two times higher in 2020 compared with 2019 across all age groups, mostly in those aged 12–24 months (6.2-fold) [19].

An Israel report investigating sociodemographic aspects evidenced an increased provenience of RSV cases from densely populated areas rather than suburbs during the unusual outbreak of 2021 [22].

The likely contributing factors might be numerous. The effect of school closures on RSV circulation is controversial. Data from the United States, where active RSV surveillance is in place, showed that re-opening schools was not associated with an increase in RSV infections [27]. Other researchers associated reopening schools and the return to in-person activities with an absolute increase of RSV activity [28]. This evidence was confirmed by Li, stating that the risk of RSV rebound in the 10 weeks that followed the full reopening of schools was significantly increased [29]. By contrast, Casalegno et al. underlines that the reduced circulation of RSV was independent from schools in France and more likely related to NPI [23].

A limitation of the review concerns the quality of reports included in the revision. Even if they all regard inpatient pediatric patients, the inclusion criteria are different because in some cases, they are limited to 18 years, and in others, to younger ages. Moreover, we must be careful when interpreting these data because they come from different continents and settings, ranging from single-site studies to multicenter ones. For the above reasons, statistical analysis was skipped.

## 5. Conclusions

The role of SARS-CoV-2 in the circulation of RSV is of interest for the consequences on health care needs and hospitalization. An unprecedented low incidence of RSV and reduced hospitalization and a subsequent out-of-season increase of the cases has been evidenced as a global phenomenon. The description of this epidemiological behavior of RSV outside of typical season cycle warrants the maintenance of strong surveillance towards the viral environment. In particular, it may be helpful to promote epidemiological surveillance throughout the year, irrespective of the expected peaks, for the rapid detection of any increase in the number of cases and potentially for adapting the pressure on healthcare systems and planning for an increase in hospital burden. Going forward, with the general population mostly vaccinated and without the severe restrictions imposed the previous year, it will be of interest to see how RSV circulation will be in the next months. Implementing mandatory NPI each winter to prevent the circulation of RSV is not a realistic option. Additional efforts should be made to potentiate prevention. The future availability of monoclonal antibodies, as well as of other preventive measures, may be of help in reducing the burden and the severity of RSV infection among pediatric patients [2,30].

## Figures and Tables

**Table 1:** Main findings of the reports included in the revision.

Reference	Country (Continent)	Type of Publication	Population	Highlighted
Kim et al., 2022 [7]	Korea (Asia)	Multicenter retrospective study	627 patients aged less than 19 years	Among RSV B cases, 169 were identified in 2017/2018, 274 in 2019/2020, and 115 in 2021/2022. No RSV cases were reported in 2020/2021. The peak circulation of RSV B epidemic was delayed by 2 months in 2021, compared with those in the pre-COVID-19 period. Among hospitalized children with RSV B infection, 32.5% required respiratory support in the 2021/2022 season, whereas 14.7% in 2017/2018 and 19.4% in 2019/2022 required respiratory support (*p* = 0.008). Analyzing by age group, infants aged 3 to 24 months had a higher rate of respiratory assistance in 2021/2022 (19.2%) than in previous seasons (1.8% in 2017/2018 and 6.0% in 2019/2020, *p* = 0.012).
Movva et al., 2022 [8]	USA (America)	Multicenter retrospective study	233 infants less than 1 year of age	In pre-COVID-19, the highest RSV proportion was observed in December–January, whereas the peaks during COVID-19 were seen in July–September. The PICU admission was higher compared to the pre-COVID 19 period (69% vs. 60%).
Bermudez Barrezueta L et al., 2022 [9]	Spain (Europe)	Observational study	270 RSV bronchiolitis less than 2 years of age	Compared to the pre-COVID-19 season, in the pandemic period, the average of 42 admissions per season was reduced to 0 in 2020/2021 season, followed by a delated and small outbreak with 17 cases for the season. A higher percentage of prematurity and PICU admissions was noted.
Dolores A et al., 2022 [10]	Argentina (America)	Observational Study	584 pediatric patients less than 12 years of age	No RSV hospitalized pediatric patient was registered in Buenos Aires during 2020; however, RSV reemerged in 2021 with a lower number of cases and a delayed outbreak. The highest age distribution did not change (<1 year).
Nenna R et al., 2022 [11]	Italy (Europe)	Observational Study	215 pediatric patients less than 18 years	Hospitalizations for acute respiratory tract infections were 82.2% lower in 2020–2021 than 2016–2017. RSV in the pre-epidemic period was identified in 130 out of 476 patients (27.3%) and in the pandemic season in 4 out 85 (4.7%).
Guitart C et al., 2022 [12]	Spain (Europe)	Prospective and observational study	782 pediatric patients	A reduced hospitalization in the post-pandemic season compared to pre-COVD-19 season (733 vs. 49) was noted.
Reyes Dominguez AI et al., 2022 [13]	Spain (Europe)	Observational study	1463 patients less than 2 years of age	A rebound in the RSV epidemic started with a six-to-nine-month delay compared to the typical season. There was a reduction in the rates of admissions for RSV acute bronchiolitis of –1.4 per thousand, but with a trend to increase 6 months after the usual fall and winter season.
Lee Cy et al., 2022 [14]	Taiwan (Asia)	Observational study	128 pediatric patients	Compared to 2014–2017 seasons, during the 2020–2021 season, patients with RSV infection were older (24.5 vs. 13.2 months, *p* < 0.001), with a higher proportion of patients aged >2 years (41/80 vs. 7/48, *p* < 0.001) and less of a need for supplementary oxygen (*p* < 0.001).
Cooney HC et al., 2022 [15]	Australia (Oceania)	Observational study	Pediatric patients aged 0–18 years	The lockdown period in Melbourne from 25 March until December 2020 reflected in no RSV admissions, followed by an unusual outbreak in January–March 2021.
Torres-Fernandez et al., 2021 [16]	Spain (Europe)	Multicenter observational study	Pediatric patients less than 2 years of age	The monthly median number of RSV hospitalization decreased from 59 in 2018 to 0.5 in 2020 and the PICU admission from 6 to 0. A 7-month delay in RSV circulation was noted after the relaxation of restrictive measures.
Hatter L et al., 2021 [17]	New Zealand (Oceania)	Epidemiological study	866 pediatric patients aged 0-4 years	In 2021, there was an incidence rate of 284 per 100 000 children aged 0–4 years, which was three times higher than the average of peaks in 2015–2019. A similar increase was seen in PICU, with an incidence rate of 15 per 100,000 children aged 0–4 years, 2.8 times higher than the average of peaks in 2015–19.
Li L et al., 2021 [18]	China (Asia)	Observational study	853 patients less than 14 years	RSV incidence increased during the COVID-19 period (September–December 2020) compared to the pre-pandemic period (September–December 2019) among hospitalized children for acute respiratory infection (20.1% vs. 6.6%).
Foley DA et al., 2022 [19]	Australia (Oceania)	Observational study	899 pediatric patients	The peak months were different for 2019 (July) and 2020 (December). The total number of RSV-positive admissions in December 2020 was more than 2.5-fold that of July 2019. In 2020, the age-specific incidence was higher (1–2 years); infants in 2020 were more likely to receive low-flow oxygen and less likely to receive pressure support at admission; hospitalization for RSV-positive bronchiolitis was significantly shorter in 2020 (2.1 days vs. 2.7 days).
Fourgeaud J et al., 2021 [20]	France (Europe)	Observational study	653 pediatric patients	No case of RSV-associated infection requiring hospitalization was observed between April and November 2020. An interseason RSV epidemic began after the end of the second national lockdown. Those admitted during the 2020/2021 interseason epidemic were more frequently aged 6 to 11 months (25.8% vs. 13.1%) and less frequently aged less than 6 months (41.3% vs. 56.6%) compared to the 2018/2019, 2019/2020 seasons.
Maruo J et al., 2022 [21]	Japan (Asia)	Retrospective observational study	193 patients aged 0–14 years	Admission for month for RSV in 2020 was 0 patients versus 4 in previous years (201–2019).
Weinberger Opek M et al., 2021 [22]	Israel (Asia)	Observational study	Pediatric patients	Absence of cases in 2020 and a rebound of cases (70 RSV cases) during spring/summer 2021 was noted. Compared to 2018–2019, a higher incidence in more densely populated areas was noted, but no differences in age, comorbidities, clinical presentation, or disease severity.
Casalegno JS et al., 2021 [23]	France (Europe)	Observational study	3415 patients less than 1 year	A reduction and a 4-month delay with no timely correlation with any major non-pharmaceutical interventions was observed in the 2020/2021 outbreak. The median age of children was increased compared to previous seasons: 4.8 months in 2020/21 compared with 2.2 to 3.1 months in the seasons 2016/17–2019/20.
Trenholme A et al., 2021 [24]	New Zealand (Oceania)	Retrospective observational study	5248 children <2 years of age hospitalized for lower respiratory tract infection (LRTI)	In 2020, there was a reduction of RSV hospitalization (2/159 LRTI) compared to previous seasons (2015: 214/1249; 2016: 224/881; 2017: 317/1012; 2018: 204/916; 2019: 388/1031).

## Data Availability

Not applicable.

## Abbreviations

RSV	Respiratory syncytial virus
PICU	Pediatric intensive care unit
LRTI	Lower respiratory tract infection
